# Transcriptome and Metabolome Analyses of *Codonopsis convolvulacea* Kurz Tuber, Stem, and Leaf Reveal the Presence of Important Metabolites and Key Pathways Controlling Their Biosynthesis

**DOI:** 10.3389/fgene.2022.884224

**Published:** 2022-07-25

**Authors:** Fang Yuan, Xiu Yin, Kaihui Zhao, Xiaozhong Lan

**Affiliations:** The Provincial and Ministerial Co-founded Collaborative Innovation Center for R&D in Tibet Characteristic Agricultural and Animal Husbandry Resources, The Center for Xizang Chinese (Tibetan) Medicine Resource, Joint Laboratory for Tibetan Materia Medica Resources Scientific Protection and Utilization Research of Tibetan Medical Research Center of Tibet, Tibet Agriculture and Animal Husbandry University, Nyingchi, China

**Keywords:** gene expression, medicinal plant, metabolites, biosynthesis pathways, tissues

## Abstract

*Codonopsis* *convolvulacea* Kurz. var. vinciflora (Kom.) L.T. Shen is a member of Campanulaceae, which is used in traditional Chinese medicine. However, apart from a few *Codonopsis* species, no detailed knowledge is available on the metabolite composition and respective transcriptome signatures. We performed a combined transcriptome and metabolome analysis of the tuber, stem, and leaf of *C. convolvulacea* and found 1,144 metabolites and 231,840 unigenes in three experimental groups. The analysis revealed considerable variations in the three tissues. Tubers were rich in amino acids and derivatives, flavonoids, and organic acids, whereas the stems and leaves were rich in alkaloids and flavonoids, respectively. Transcriptome sequencing revealed candidate genes being involved in flavonoid, tryptophan, and alkaloid biosyntheses. In particular, we indicated that the variation in the isoflavone content is linked to the expressions of CHI, CYP73A, C3′H, F3H, CYP75B1, anthocyanidin synthase, and FLS. In a similar way, the levels of indole, L-tyrosine, and tryptamine were also consistent with the expressions of TDC/DDCs in the respective tissues. In addition, the expression levels of ASP5, ARO8, GOT, and AOC3 indicated that L-tryptophan is being converted to downstream metabolites. Overall, our datasets present a useful resource for future research on the uses of this medicinal plant and put forward many research questions.

## 1 Introduction

*Codonopsis* is the largest genus in the family Campanulaceae with more than 40 species. These species are distributed in Asia; around 39 species are distributed in China (Hong, 2015). Members of this genus have long been used in traditional Tibetan medicine and are described in the Chinese pharmacopoeia. The most famous *Codonopsis* species include *Codonopsis pilosula*, *C. lanceolata*, *C. Tangshan*, *C. clematidea, C. cordifolioidea*, *C. nervosa*, *C. thalictrifolia*, *C. xundianensis*, and *C. tubulosa*, which have been extensively explored for their phytochemistry, and >100 compounds have been isolated and identified (He et al., 2015). Phytochemical investigations have shown the accumulation of polyacetylenes, polysaccharides, alkaloids, coumarins, lignans, flavonoids, phenylpropanoids, organic acids, essential oils, and terpenoids. These compounds have been extracted from various parts such as aerial parts (stem and leaves) and roots/tubers; for example, phenylpropanoids were reported to occur in the aerial parts of *C. nervosa* and *C. Tangshan* (Tsai and Lin, 2008; Xie G et al., 2011) and in the roots of *C. lanceolata* (Ren et al., 2013) and *C. cordifolioidea* (Hu et al., 2012). Some studies have also focused on the chemotaxonomical identification, purification, and characterization of useful chemicals from this genus (Wang et al., 1995). However, not all the species have been explored in terms of their phytochemistry and molecular mechanisms governing the particular biochemical composition. Studies involving bioactivities of the *Codonopsis* extracts have only been reported in a limited number of species, i.e., *C. pilosula, C. lanceolata, C. clematidea*, and *C. cordifolioidea.* Extracts from *C. pilosula* and *C. lanceolata* have also shown antitumor activity (Wang et al., 1995), antidiabetic (Fu et al., 2008), and antiaging effects (Xu et al., 2006), as well as a protective effect against gastric mucosal damage (Liang and Jianhua, 1989), inhibition of erythrocyte hemolysis (Ng et al., 2004), enhancement of the nerve growth factor (Liu et al., 2003), attenuated angiotensin II (AngII)-induced insulin-like growth factor II receptor promoter activity (Tsai et al., 2013), and hepatoprotective activity (Liang et al., 2007). Detailed exploration of *Codonopsis* species lags because they are found mostly in the wild in selected regions, which makes it difficult to collect them.

*C convolvulacea* Kurz. var. vinciflora (Kom.) L.T. Shen (Flora of China; http://www.efloras.org/florataxon.aspx?flora_id=2&taxon_id=200022897) (Zhang et al., 2015) is one such species that has not been explored in detail. It is naturally distributed in the Northwest Yunnan and Tibetan plateau of China (including the adjacent region of Dolpo, Nepal) (Ghimire and Aumeeruddy-Thomas, 2009; Chen et al., 2021). The *C. convolvulacea* plant has a short stem base, and few tumor-like scars can be seen on the stem. The leaves are alternate or sometimes opposite; the petioles are obvious, up to 1.6 cm long, the leaves are thin, membranous, the edges are toothed, and the veins are thin and obvious. The tubers are massive, nearly ovoid or egg-shaped. In the past, it was used to treat chest pain and cold and to relieve appetite-related issues. Furthermore, in Tibetan medicine, it is used for the treatment of coronary heart diseases, mountain sickness, strengthening of the spleen and stomach, and multiple other diseases (Tang et al., 2017 and references therein). In Tibetan medicine, it has been reported as a treatment for diabetes mellitus (Gao et al., 2021). It has also been reported that some subspecies of *C. convolvulacea*, e.g., subsp. Forrestii, is used to treat eye diseases as well as used as a functional food by stewing with meat and streaming with egg. A very basic level study revealed that *C. convolvulacea* Kurz. has 17 different types of amino acids, proteins, carbohydrates, vitamins, fiber, β-carotenoids, and mineral elements (Zhang et al., 2015). Another study on *C. convolvulacea* roots reported the extraction of five phenolic extracts, i.e., taraxerone, taraxerol, shikimic acid, syringaresinol, and stigmasterol (Guang-Xuan et al., 2001).

Developments in omics technologies have enabled researchers to understand the chemical composition of plants as a whole and/or plant parts on a global scale. Next-generation sequencing has significantly helped to understand the possible pathways and genes that govern the biosynthesis and accumulation of valuable compounds in plants of interest. In the case of *Codonopsis,* only limited studies have been conducted to understand the metabolome and transcriptome profiles. An untargeted metabolome profiling of the roots of *C. pilosula* reported the presence of carboxylic acids, steroids, organic acids, and flavonoids (Zeng et al., 2021) and revealed that stems and leaves are richer in polyacetylenes, flavonoids, alkaloids, and organic acids; these parts were previously considered waste materials. Thus, this report opens new possible raw materials for the extraction of such compounds in higher quantities. Transcriptome sequencing of *C. pilosula* helped in the identification of the candidate genes that are involved in polysaccharide biosynthesis (Gao et al., 2015). However, these studies provide very limited information on the global metabolomic profile of *C. convolvulacea.* In particular, it is important to understand the types of metabolites that accumulate in the three major organs, i.e., tubers, shoots, and leaves. In addition, the transcriptome sequencing will be very much helpful in understanding the key pathways as well as putative major genes that can affect the accumulation of these metabolites. Here, we conducted a pioneering combined metabolome profiling and transcriptome sequencing of tubers, stems, and leaves of *C. convolvulacea*. We discuss the metabolome profiles of *C. convolvulacea* tubers, stems, and leaves according to the respective biosynthesis pathways. These results provide bases for the use of different plant parts in traditional Chinese herbal medicine.

## 2 Results

### 2.1 Metabolome Profile of *C. convolvulacea* Tubers, Shoots, and Leaves

**FIGURE 1:**
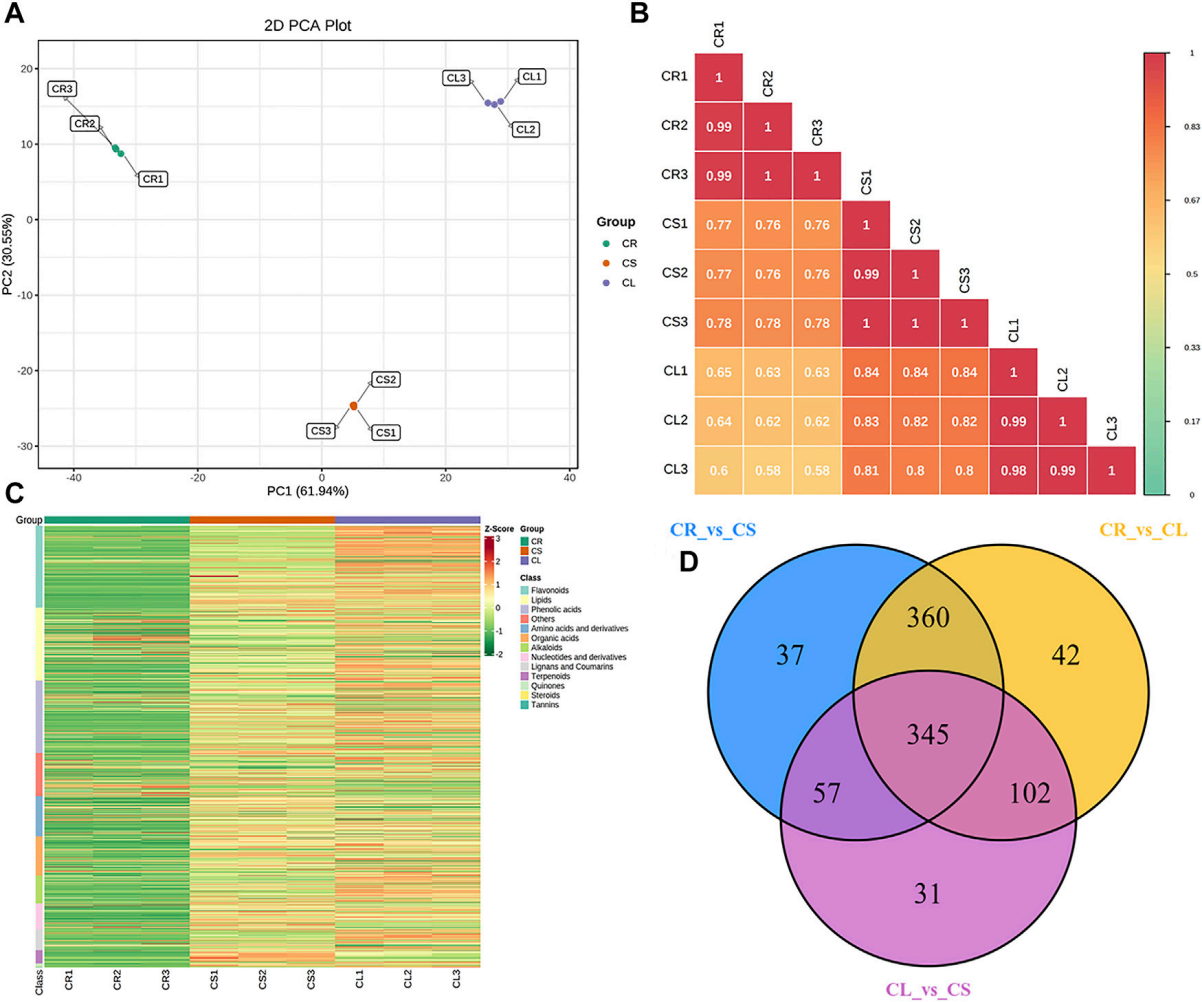
Metabolome analysis of tuber, stem, and leaves of *C. convolvulacea.* **(A)** Principal component analysis, **(B)** Pearson’s correlation coefficient, **(C)** heatmap of the metabolite concentrations and their classification, and **(D)** venn diagram representing the number of metabolites that were differentially accumulated in the three comparisons. CS, CL, and CR represent stem, leaf, and tuber, respectively.

With the untargeted metabolomics approach using the UPLC-MS/MS detection platform, we detected a total of 1,144 metabolites in three experimental groups (Supplementary Tables S1–3). The principal component analysis (PCA) showed a segregating trend between the three samples (Figure 1A). Overall, there was 61.94% and 30.55% variation present on PC1 and PC2, respectively. A relatively higher Pearson’s correlation coefficient (PCC) was recorded between the replicates of each tissue type, i.e., tuber, stem, and leaf. A higher PCC was observed between CL and CS (0.82), followed by CS and CR (0.77), and CL and CR (0.62) (Figure 1B). This indicates that the metabolites accumulated in leaves and stems might be similar to some extent and could be different from those of tubers. The diverse set of detected metabolites could be grouped into thirteen different classes (Figure 1C). The metabolites were screened by combining the *p*-value or the fold change values. We considered the compounds as differentially accumulated metabolites (DAMs) if the variable importance in projection (VIP) was ≥1. Overall, we observed 849, 799, and 535 DAMs between CR vs. CL, CR vs. CS, and CL vs. CS, respectively. Of these, 345 metabolites were common among all the tissues (Figure 1D).

### 2.2 Differential Metabolome Profiles

**FIGURE 2:**
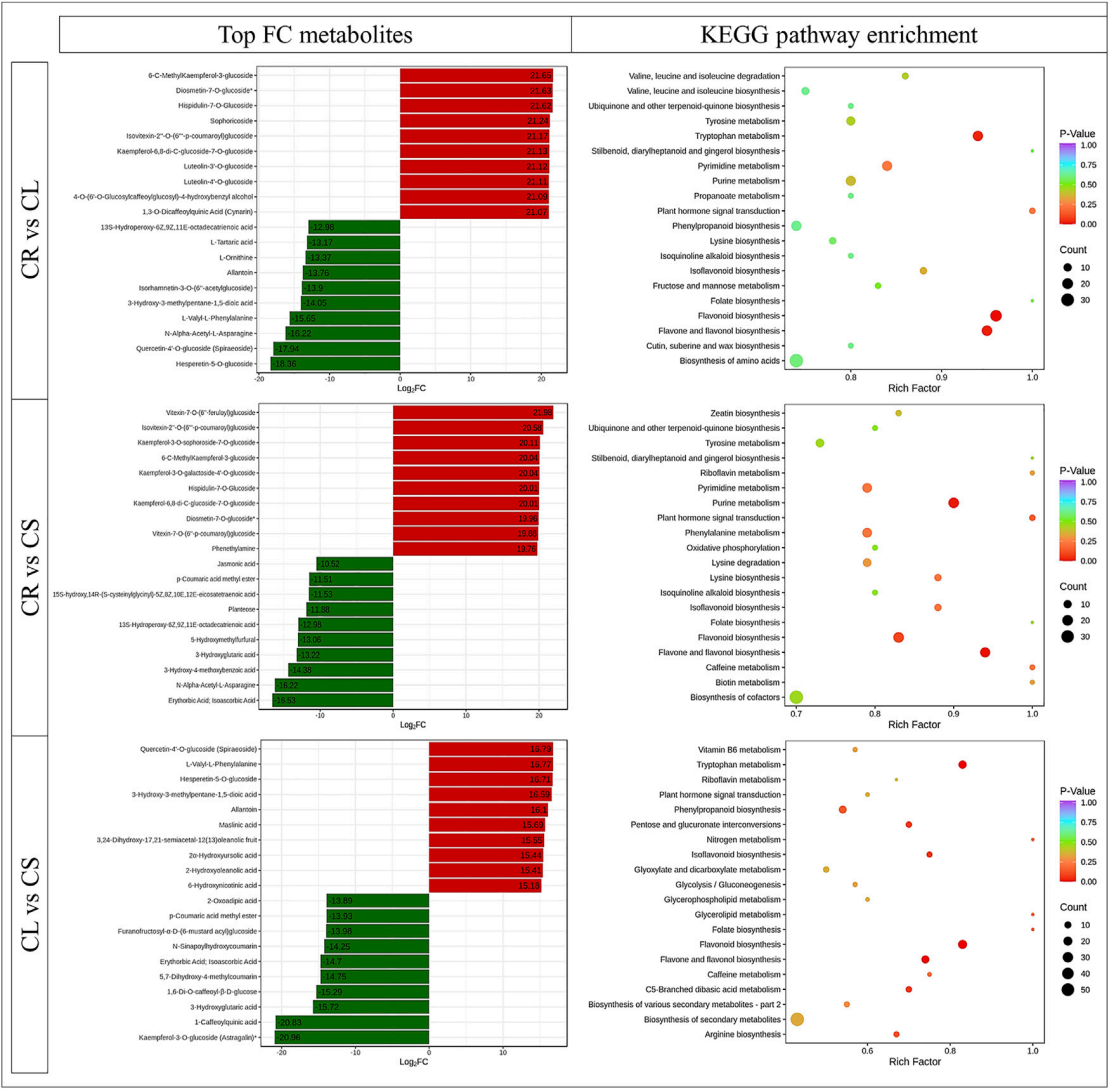
Top-accumulated metabolites in different *C. convolvulacea* tissues (CL, leaf; CS, stem; CR, tuber) and KEGG scatter plots showing pathways to which the DAMs were enriched.

The highest 10 accumulated metabolites in CR belonged to three classes, i.e., amino acids and derivatives, flavonoids, and organic acids. In contrast, eight of the highest 10 accumulated metabolites in CL were flavonoids, whereas the other two were phenolic acids (Figure 2). The DAMs between CR and CL were enriched in flavonoid biosynthesis, flavone and flavonol biosynthesis, and tryptophan metabolism pathways. When we compared the DAMs between CR and CS, the top 10 most accumulated metabolites belonged to amino acids and derivatives, free fatty acids, organic acids, phenolic acids, saccharides, alcohols, and vitamins. In contrast, alkaloids were the top accumulated group in CS. The DAMs between CR vs. CS were enriched in flavonoid biosynthesis, flavone and flavonol biosynthesis, and the purine metabolism pathway (Figure 2). Flavonoids, lignans and coumarins, organic acids, and phenolic acids were the most enriched metabolites in CL whereas, in CS, alkaloids, terpenoids, organic acids, amino acids and derivatives, and flavonoids were the top 10 accumulated metabolites as compared to CL. The DAMs were enriched in the same pathways as CR vs. CL. In addition, phenylpropanoid biosynthesis, isoflavonoid biosynthesis, and arginine biosynthesis pathways were enriched (Figure 2).

We searched for tissue-specific metabolites within every three comparisons, i.e., CR vs. CS, CR vs. CL, and CS vs. CL, and generated a Venn diagram (Supplementary Figure S1). Of these tissue-specific accumulated metabolites, 322, 8, and 27 were common between CS and CL, CL and CR, and CR and CS, respectively. There were 14, 33, and 4 metabolites specific to CS, CL, and CR, respectively. The 33 CL-specific metabolites were classified as lignins and coumarins, phenolic acids, and alkaloids. Apart from these metabolites, we studied the pathways to which DAMs were significantly enriched. In particular, we focused on the common and unique pathways within each three-tissue comparison. The details are provided as follows.

#### 2.2.1 Flavonoid Biosynthesis

**FIGURE 3:**
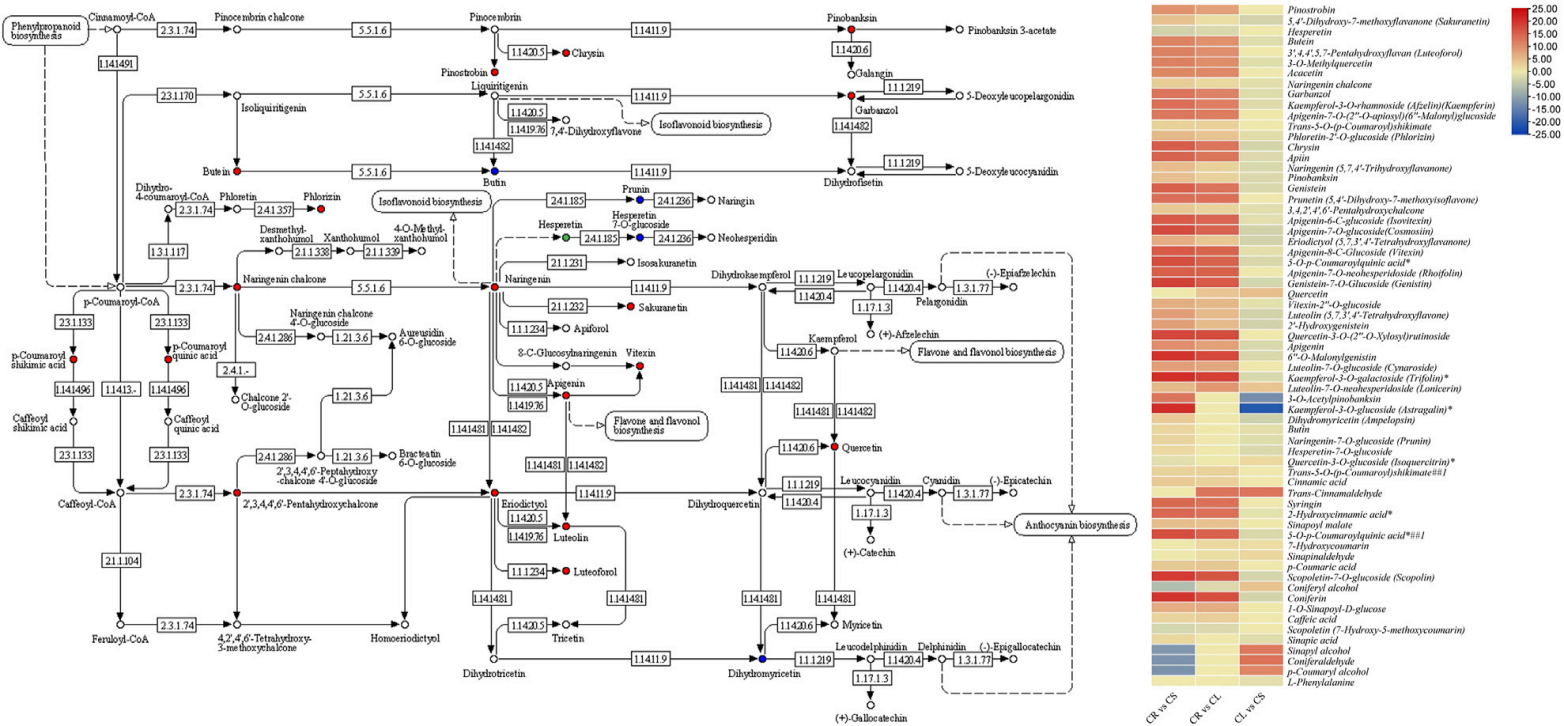
Flavonoid biosynthesis pathway showing enriched DAMs between CS and CL. The heatmap on the right shows the log2 fold change values of the DAMs enriched in the flavonoid biosynthesis pathway in the three tissue comparisons. CL, leaf; CS, stem; CR, tuber.

Forty-four DAMs were enriched in flavonoid biosynthesis-related pathways, e.g., flavonoid biosynthesis, flavone and flavonol biosynthesis, and isoflavonoid biosynthesis pathways. Apart from these 44 DAMs, 21 metabolites were also enriched in the phenylpropanoid biosynthesis pathway, which is present upstream of the flavonoid biosynthesis pathways (Figure 3). Of the 21, L-phenylalanine was not detected in CR, whereas its accumulation was higher in leaves as compared to the stem. Going down the pathway, we observed that four phenolic acids, i.e., trans-cinnamaldehyde, sinapyl alcohol, coniferaldehyde, and p-coumarin alcohol, were not detected in leaves while they were present in elevated levels in CS as compared to CR. Most of the other phenolic acid levels were higher in CS as compared to CR but lower in CL as compared to CR. Lignans and coumarins had higher accumulation in CS as compared to CR and CL indicating a higher cellulosic content in the stem as compared to tubers and leaves. Upstream the flavonoid biosynthesis pathway, p-coumaric acid is present, whose level was higher in CS (log2 foldchange = 3.67) and CL (log2 foldchange = 3.80). Within the flavonoid biosynthesis pathway, we observed that all the metabolites except two intermediates (hesperetin and quercetin-3-O-glucoside) had higher accumulation in CL and CS as compared to CR. Of these, 31 flavonoids had higher quantities detected in CL as compared to CS. Interestingly, all the metabolites enriched in the isoflavonoid biosynthesis pathway also showed a similar accumulation trend, i.e., the highest concentrations were measured in CL followed by CS and CR. Overall, these observations indicate that 1) leaves are the most appropriate source of flavonoids, phenolic acids, and isoflavonoids followed by stems and tubers. 2) The differential accumulation of these metabolites in the studied tissues is being regulated at various levels, i.e., higher initial levels of L-phenylalanine as well as other steps in the three pathways, i.e., phenylpropanoid biosynthesis, flavonoid biosynthesis, and isoflavonoid biosynthesis pathways.

#### 2.2.2 Tryptophan Metabolism

**FIGURE 4:**
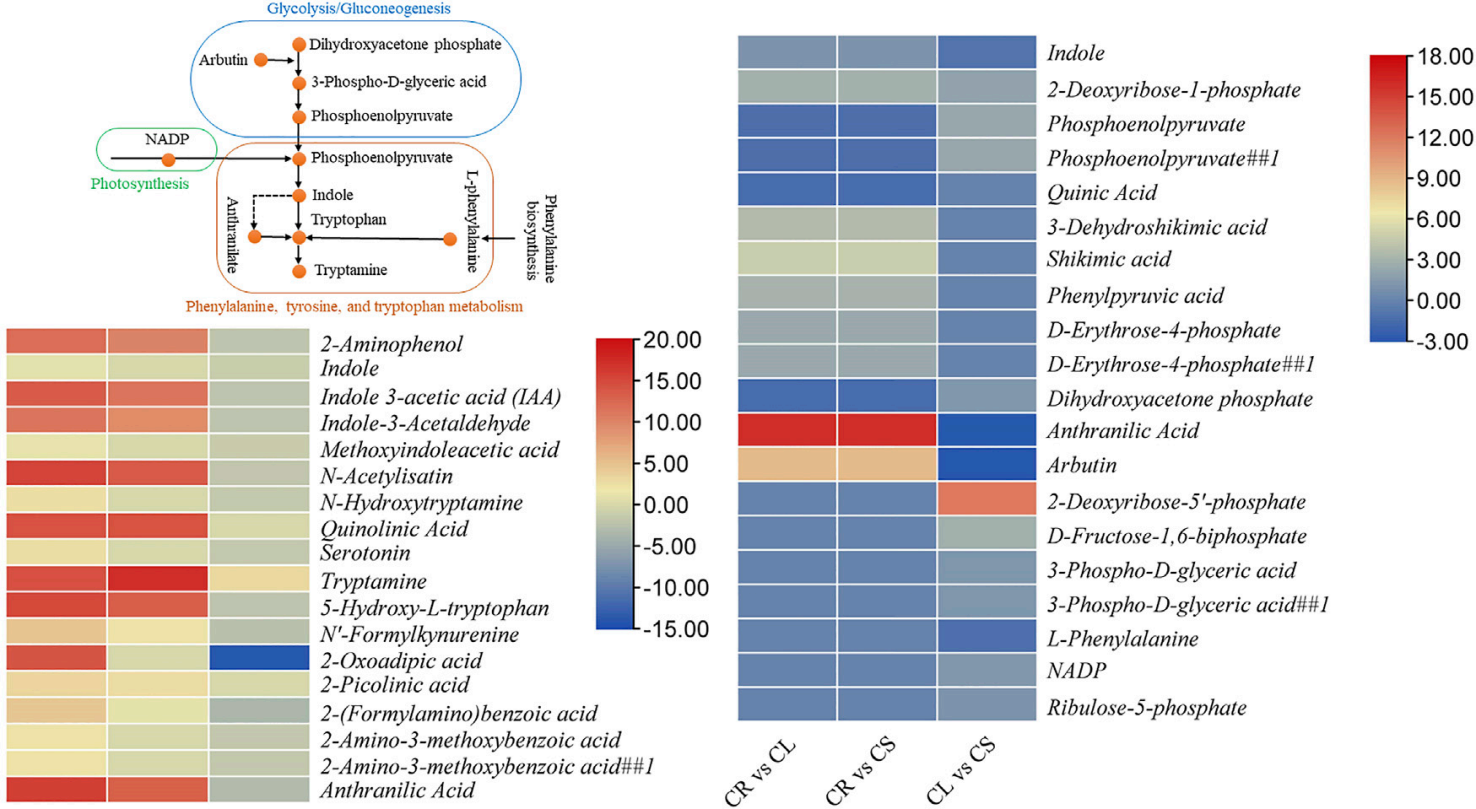
Differential regulation of tryptophan metabolism pathway in *C. convolvulacea* leaf (CL), stem (CS), and tuber (CR). The heatmaps show the metabolites that were enriched in tryptophan metabolism and/or the upstream pathways.

Tryptophan has important functions within plants as well as is considered an important compound that is involved in the secretion of melatonin and serotonin in human bodies. These compounds regulate the sleep–wake cycle, appetite, mood, and pain. Furthermore, it can be used to produce niacin, i.e., vitamin B3, which is needed for DNA production and energy metabolites. The comparative metabolome profiles of CR, CL, and CS indicate that 18 DAMs were enriched in tryptophan metabolism in at least one comparison. All the DAMs had higher accumulation in CL followed by CS and CR. Seven of these metabolites were not differentially accumulated between CR ad CS (Figure 4). Of particular interest was the highest accumulation of indole in leaves followed by stem and tubers, although it was not differentially accumulated between CR and CS, indicating similar levels in these two tissues. To fully understand this elevated accumulation in leaves, we checked the accumulation patterns of the metabolites present in the upstream pathways such as phenylalanine, tyrosine, and tryptophan biosynthesis, photosynthesis, glycolysis/gluconeogenesis, and pentose phosphate pathway. In the case of photosynthesis, only one metabolite was found to be differentially accumulated between the leaves and stem, i.e., NADP (nicotinamide adenine dinucleotide phosphate). Moreover, it was only detected in CL, thus providing a molecule for higher downstream metabolite biosynthesis in CL. Furthermore, arbutin (enriched in glycolysis/gluconeogenesis) was also accumulated in higher quantities in CL as compared to CR and CS, respectively. However, the metabolites enriched in the pentose phosphate pathway were highly accumulated in CS followed by CL and CR, indicating a limited role for the higher downstream pathways. At last, three of the five metabolites enriched in phenylalanine, tyrosine, and tryptophan biosynthesis, i.e., indole, anthranilic acid, and L-phenylalanine were accumulated in higher quantities in CL as compared to CS (Figure 4). These differential accumulations indicate that CS could be a rich source of tryptophan or its derivative, i.e., tryptamine, followed by CL.

#### 2.2.3 Other Important Pathways

We further studied pathways such as indole alkaloid biosynthesis, stilbenoid, diarylheptanoid, and gingerol biosynthesis, terpenoid backbone biosynthesis, ubiquinone and other terpenoid–quinone biosynthesis, tropane, piperidine, and pyridine alkaloid biosynthesis, and isoquinoline alkaloid biosynthesis pathways. These pathways are important for the biosynthesis of active compounds and are important for medication purposes. Altogether, 19 metabolites were enriched in these pathways. Except for L-lysine, all metabolites were accumulated in higher quantities in CL and CS as compared to CR. L-Tyramine, L-phenylalanine, mevalonic acid, 4-hydroxybenzoic acid, 5-O-p-coumaroylquinic acid, and protocatechualdehyde were accumulated in higher quantities in leaves as compared to the stem. In contrast, tryptamine, L-lysine, and homogentisic acid were accumulated in higher quantities in CS as compared to CL (Table 1). These changes are consistent with the other observations that CL is richer in flavonoids as compared to CS and CL.

**TABLE 1:** Log2 foldchange values of the differentially accumulated metabolites in *C. convolvulacea* leaf (CL), stem (CS), and tuber (CR) enriched in important KEGG pathways.

Metabolite	Class	CR vs. CL	CR vs. CS	CL vs. CS
Indole alkaloid biosynthesis				
Tryptamine	Alkaloids	14.37	17.46	3.08
Isoquinoline alkaloid biosynthesis				
L-Tyramine	Alkaloids	16.42	15.34	−1.07
3,4-Dihydroxy-L-phenylalanine (L-Dopa)	Amino acids and derivatives	2.46	2.00	0
p-Coumaric acid	Phenolic acids	3.66	3.80	0
Protocatechualdehyde	Phenolic acids	2.72	1.18	−1.53
Stilbenoid, diarylheptanoid, and gingerol biosynthesis				
5-O-p-Coumaroylquinic acid	Phenolic acids	17.26	14.96	−2.29
Trans-5-O-(p-Coumaroyl)shikimate	Phenolic acids	2.67	2.57	0
Terpenoid backbone biosynthesis				
Mevalonic acid	Organic acids	15.58	14.31	−1.26
Tropane, piperidine, and pyridine alkaloid biosynthesis				
Piperidine	Alkaloids	1.07	0	0
Allysine (6-Oxo DL-Norleucine)	Amino acids and derivatives	3.07	2.40	0
L-Isoleucine	Amino acids and derivatives	1.04	1.02	0
L-Lysine	Amino acids and derivatives	−2.83	−1.35	1.47
L-Phenylalanine	Amino acids and derivatives	0	0	−1.21
Phenylpyruvic acid	Organic acids	3.09	3.03	0
L-Pipecolic Acid	Organic acids	0	1.04	0
Nicotinic acid (Vitamin B3)	Others	2.49	2.12	0
Ubiquinone and another terpenoid-quinone biosynthesis				
4-Hydroxybenzoic acid	Phenolic acids	3.03	1.41	−1.61
Cinnamic acid	Phenolic acids	3.31	2.47	0
Homogentisic acid	Phenolic acids	4.10	5.11	1.01

Zero means that the metabolite is not differentially accumulated between the two tissues.

### 2.3 Transcriptome Profile of *C. convolvulacea* Leaf, Stem, and Tuber

**FIGURE 5:**
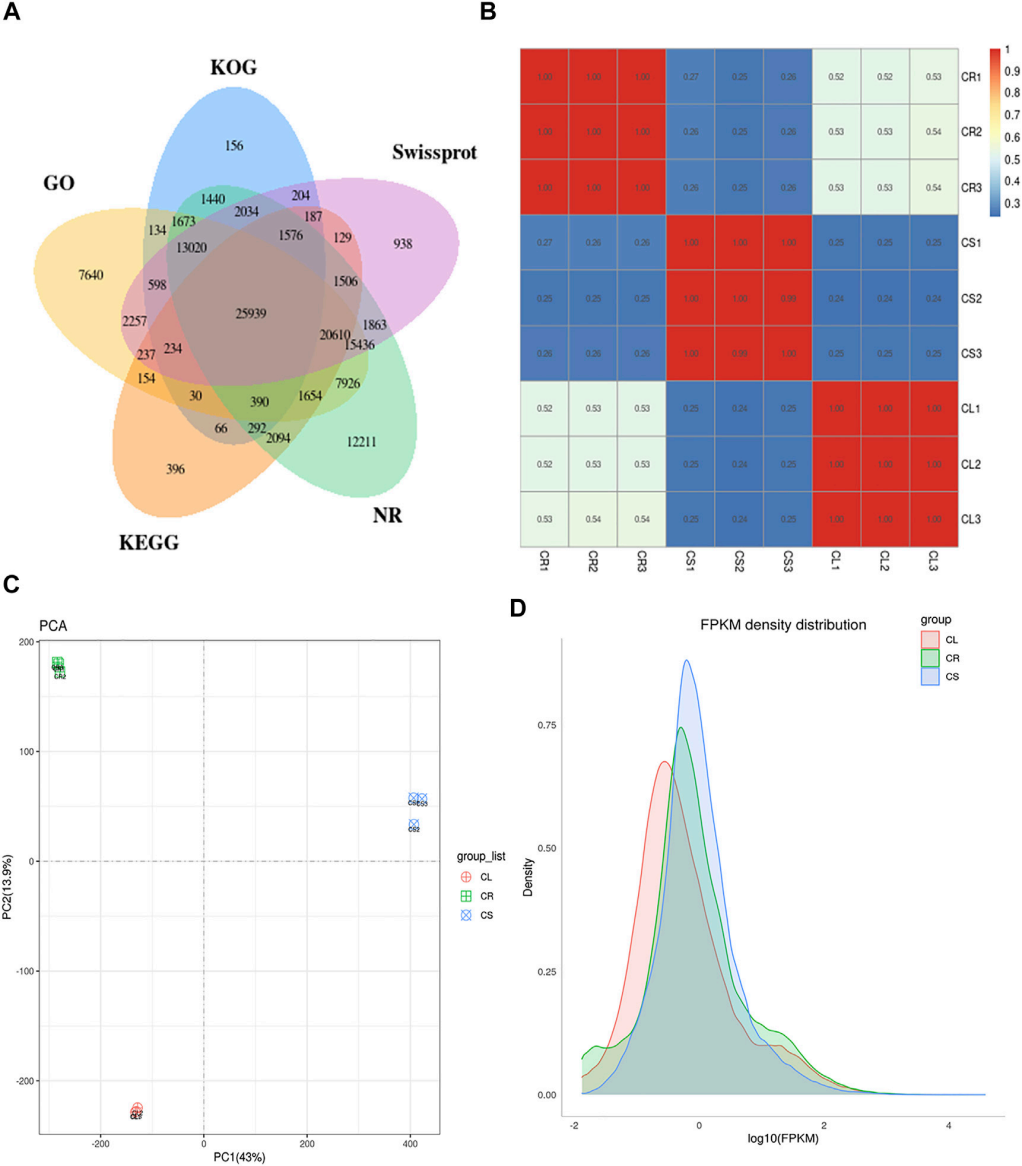
Transcriptome analysis of *C. convolvulacea* leaf (CL), stem (CS), and tuber (CR). **(A)** Venn diagram showing annotation of the unigenes in different databases, **(B)** Pearson’s correlation coefficient, **(C)** principal component analysis, and **(D)** FPKM density distribution graph.

Nine RNAseq libraries generated clean reads for each tissue ranging from 39,278,448 to 40,712,104 (average 40,174,095 reads). The average Q20 and Q30 rates were 93.96% and 97.86%, respectively. The average GC% for the libraries was 46.11% (Supplementary Table S4). Using the high-quality reads, the transcripts were assembled leading to the identification of unigenes with an average length of 748.50 bp and N50 length of 1,197 bp. In total, 123,024 unigenes could be annotated in different databases (Figure 5A). Maximum PCC based on fragments per kilobase of transcript per million fragments mapped (FPKM) values was noted between the replicates for each tissue while there was ≤0.53 indicating that the sampling was reliable (Figure 5B). PCA showed the grouping of the same tissue replicates together, whereas the PC1 and PC2 explained 43% and 13.9% variations between the samples, respectively (Figure 5C). Overall, the FPKM for CS was higher followed by CR and CL (Figure 5D).

### 2.4 Differential Gene Expression Between *C. convolvulacea* Leaf, Stem, and Tuber

**FIGURE 6:**
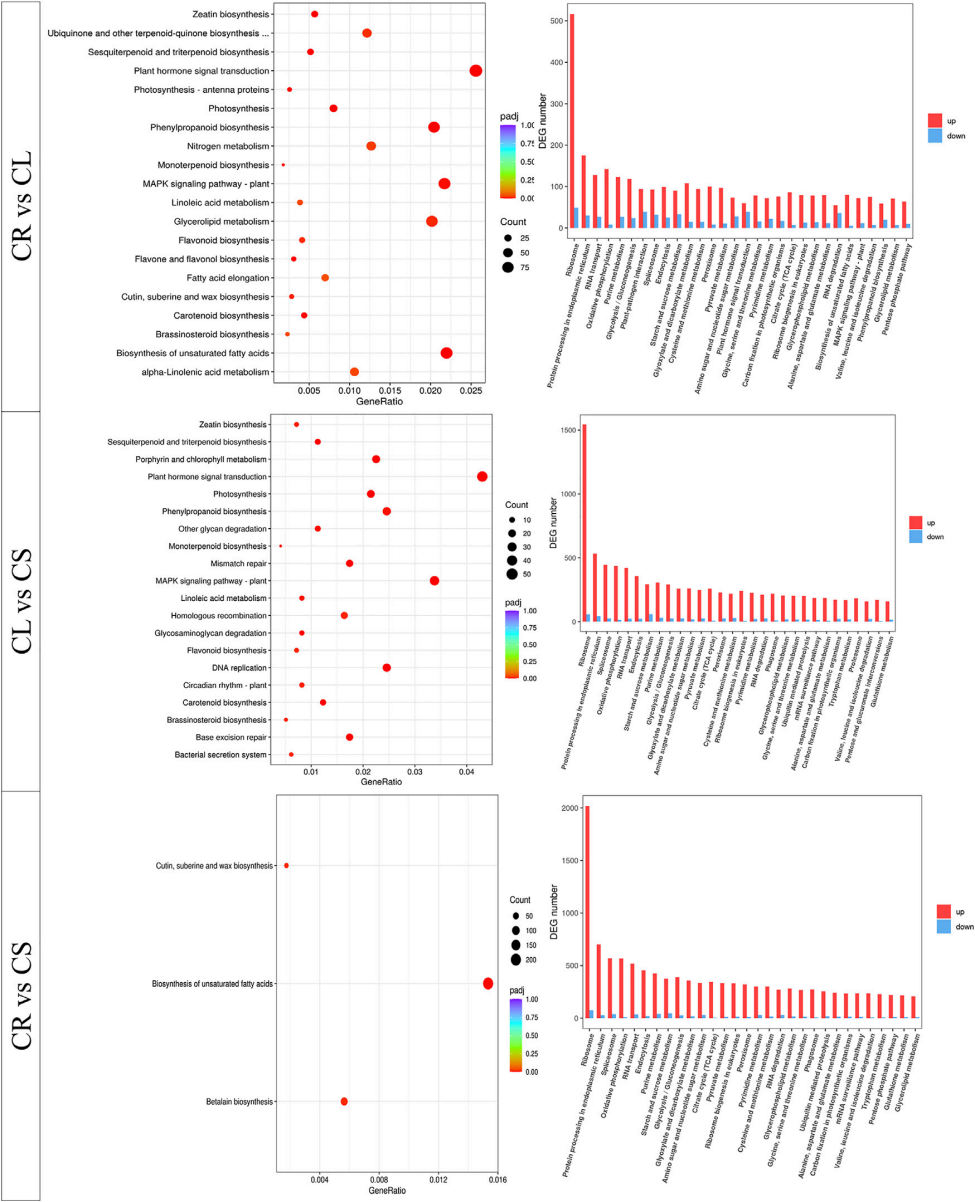
KEGG pathway enrichment analysis of the differentially expressed genes between leaf (CL), stem (CS), and tuber (CR) in *C. convolvulacea.*

The comparative transcriptome analysis revealed that 68,605, 23,606, and 50,210 transcripts were differentially expressed between CR vs. CS, CR vs. CL, and CL vs. CS, respectively (Supplementary Figure S2; Figure 6). A relatively higher number of genes were upregulated in CS, CL, and CS as compared to CR, CR, and CL, respectively (Figure 6). The KEGG classification showed that the genes could be classified into five major categories, i.e., cellular processes, environmental information processing, genetic information processing, metabolism, and organismal systems (Supplementary Figure S3A). On the other hand, GO annotation classified the annotated transcripts into three major categories, i.e., biological process, cellular components, and molecular function. Most of the genes were categorized as molecular functions (Supplementary Figure S3B).

The KEGG pathways enrichment analysis showed that the DEGs could be mapped onto different pathways. The DEGs between CR vs. CL and CL vs. CS were enriched in multiple pathways involved in signaling, growth and development, and biosynthesis of important secondary metabolites. The DEGs between CR vs. CS were enriched in the biosynthesis of unsaturated fatty acids, betalain biosynthesis, cutin submarine, and wax biosynthesis. Apart from these three pathways, DEGs were also enriched in multiple other pathways (Figure 6). The pathway-specific results are presented as follows.

#### 2.4.1 Differential Transcriptomic Changes in Flavonoid Biosynthesis

A total of 232 DEGs were enriched in flavonoid biosynthesis-related pathways, i.e., flavonoid biosynthesis (39), flavone and flavonol biosynthesis (22), and phenylpropanoid biosynthesis (171) pathways. In particular, in the flavonoid biosynthesis pathway, the differentially expressed transcripts were annotated as ten major genes (Supplementary Table S5). Of the 39, 29 genes were upregulated in CS as compared to CR, while six were upregulated in CR. The very first step in flavonoid biosynthesis is the conversion of cinnamoyl-CoA into pinocembrin chalcone by chalcone synthase (CHS) and p-coumaroyl-CoA by trans-cinnamate 4-monooxygenase (CYP73A). Both CHS and CYP73A transcripts had higher expressions in CS and CL as compared to CR; CL had lower expressions than CS. Pinocembrin is further converted into pinobanksin by naringenin 3-dioxygenase (F3H), which was upregulated in CS as compared to CR and downregulated in CL as compared to CS. Apart from these, chalcone isomerase (CHI), anthocyanidin synthase (ANS), flavonoid 3′-monooxygenase (CYP75B1), C3′H, 5-O-(4-coumaroyl)-D-quinate 3′-monooxygenase (CYP98A), HCT, shikimate O-hydroxycinnamoyltransferase, caffeoyl-CoA O-methyltransferase (CCOAOMT1), and bifunctional dihydroflavonol 4-reductase/flavanone 4-reductase (DFR) were the key genes that showed differential expression in CR vs. CS, CR vs. CL, and CS vs. CL. One important thing was the reduced expression of these genes in CL as compared to CS. In particular, three HCTs, a CYP75B1, two CHIs, and CCOAOMT1 were upregulated in CL as compared to CS. The higher expression of these genes could be a possible cause of higher isoflavone and flavonoid contents in leaves. The transcripts enriched in the phenylpropanoid biosynthesis pathway were annotated as 14 major genes (Supplementary Table S5). A higher number of transcripts were annotated as peroxidases (PODs), 4-coumarate--CoA ligase (4CL), and beta-glucosidase (bglX). A total of 135 and 59 genes showed higher expressions in CS and CL as compared to CR, respectively. We found that four PODs, a CCOAOMT, eleven bgIXs, three 4CLs, a UGT72E (coniferyl-alcohol glucosyltransferase), three HCTs, and a cinnamyl-alcohol dehydrogenase showed higher expressions in CL as compared to CS, indicating that these genes are probably causing higher accumulation of flavonoids in the CLs as compared to CS and CR. Downstream the flavonoid biosynthesis pathway, the flavone and flavonol pathway had two major genes, i.e., UGT73C and CYP75B1 which were differentially expressed between the studied tissues. Interestingly, all the transcripts annotated as UGT73C were solely expressed in CS. On the other hand, the CYP75B1s had variable expression patterns in the three tissues. A large number of CYP75B1 (10) transcripts were not differentially expressed between CS and CL (Supplementary Table S5).

#### 2.4.2 Differential Transcriptomic Changes in the Phenylalanine, Tyrosine, and Tryptophan Metabolism

Ninety-five transcripts associated with twenty genes were enriched in the phenylalanine, tyrosine, and tryptophan metabolism pathway. Of these, 87, 31, and 65 genes were differentially expressed between CR and CS, CR and CL, and CS and CL, respectively. We found that a large number of genes were solely expressed in the stem indicating that most of the changes in this pathway occur within the stem as compared to tubers. Almost all the genes that are present upstream of the indole and L-tryptophan were upregulated in CS and CL as compared to CR, strongly proposing that stems and leaves offer higher L-tryptophan contents due to the increased expression of these genes. Since the metabolome analysis had shown higher indole content, we looked for genes that could have played role in this higher accumulation in leaves. Most genes showed variable expressions in the CS vs. CL, i.e., transcripts of the same genes were both upregulated as well as downregulated. However, we found two tryptophan synthase (trpB; *TRINITY_DN3970_c0_g1_i25* and *TRINITY_DN6592_c0_g1_i27*) genes had higher expression in CL as compared to CS, while its expression was fractional in CR. These two genes are valuable for future studies where higher expressions of these can help produce higher L-tryptophan in the leaves as well as in the stem. Apart from these genes, transcripts of the other genes including 3-dehydroquinate dehydratase II (aroQ), 3-dehydroquinate dehydratase/shikimate dehydrogenase (aroDE), anthranilate synthase component I (trpE), anthranilate phosphoribosyltransferase (trpD), and indole-3-glycerol phosphate synthase (trpC) also showed relatively higher expressions in CS and CL as compared to CR. Their expressions, however, were variable in CL as compared to CS (Supplementary Table S5). Going further upstream, we found that DEGs were also significantly enriched in the photosynthesis pathway. This result is obvious since both leaves and stems are green parts, with leaves being the main sites for photosynthesis. Almost all the DEGs enriched in photosynthesis were upregulated both in CS and CL as compared to CR, with the highest expression in CL (Supplementary Table S5).

#### 2.4.3 Differential Transcriptomic Changes in Alkaloid Biosynthesis

Since the metabolome profile showed a higher accumulation of alkaloids and terpenoids in leaves as well as in stems, we explored the transcriptomic signatures related to it. In the case of the indole alkaloid biosynthesis, seven transcripts encoding aromatic-L-amino-acid/L-tryptophan decarboxylase (DDC) were found, all of which were solely expressed in CS. Most of the genes enriched in the tropane, piperidine, and pyridine alkaloid biosynthesis pathway were expressed in CL and CS, with the highest expressions in CS followed by CL and CR confirming the metabolome profile results that the top accumulated metabolites in the stem were alkaloids. Of particular interest were primary-amine oxidase (AOC3), aspartate aminotransferase (ASP5), histidinol-phosphate aminotransferase (hisC), aromatic amino acid aminotransferase I/2-aminoadipate transaminase (ARO8), and aspartate aminotransferase (GOT1 and GOT2). Twenty-three genes were enriched in sesquiterpenoid and triterpenoid biosynthesis. The expression pattern of these genes was similar to that related to the indole alkaloid biosynthesis pathway, i.e., highest expression in CS, followed by CL and CR. Notable genes were squalene monooxygenase (SQLE) and farnesyl-diphosphate farnesyltransferase (FDFT1). However, 11 of these were not expressed in CR at all. Twenty-four genes were enriched in the stilbenoid, diarylheptanoid, and gingerol biosynthesis pathways. All of these genes were expressed in CS. One important observation was that no expression of 5-O-(4-coumaroyl)-D-quinate 3′-monooxygenase (C3′H) was noted in CL. This gene converts p-coumaroyl-shikimic acid and p-coumaroyl quinic acid to caffeoyl shikimic acid and caffeoyl quinic acid, respectively (Schoch et al., 2001). These two compounds are important precursors for the biosynthesis of gingerol. Thus, this gene is an interesting target for future studies on gingerol biosynthesis in this species. Interestingly, most of the genes enriched in this pathway were downregulated in leaves when compared to tubers and stems, indicating that leaves have limited quantities of phenolic compounds, which is consistent with the metabolome profiling. In ubiquinone and other terpenoid–quinone biosynthesis pathways, 124 DEGs were enriched. A large number of transcripts were annotated as 4-hydroxyphenylpyruvate dioxygenase (HPD) (16), 4-coumarate--CoA ligase (4CL) (29), and NAD(P)H dehydrogenase (quinone) (wrbA) (35). Twenty-five genes including six HPDs, CYP73A, ARO8, seven 4CLs, four wrbAs, and six COQs were CS specific indicating their roles in the terpenoid–quinone biosynthesis. No expression of 83 transcripts in CR suggests that tubers are not the best source of terpenoid–quinone (Supplementary Table S5). This observation is further strengthened by no expression of all the DEGs enriched in the tropane, piperidine, and pyridine alkaloid biosynthesis pathway (Supplementary Table S5).

#### 2.4.4 Differential Transcriptomic Changes in Cutin, Suberin, and Wax Biosynthesis, Biosynthesis of Unsaturated Fatty Acids, and Betalain Biosynthesis

One important observation was the enriched cutin, suberin, and wax biosynthesis as well as biosynthesis of unsaturated fatty acids and betalain biosynthesis pathways in CR vs. CS comparison. Only one gene (TDC, aromatic-L-amino-acid/L-tryptophan decarboxylase) was enriched in the betalain biosynthesis pathway. This gene was upregulated in CL as compared to CS, whereas it was not expressed in CR at all. In a similar manner, the six genes enriched in the biosynthesis of unsaturated fatty acids were only differently expressed between CS and CL; CL had higher expression of these genes as compared to CS. The third pathway (cutin, suberin, and wax biosynthesis) also showed a similar expression trend, i.e., higher expression in CL followed by CS and CR. Overall, these expression patterns indicate that leaves have a higher potential for the biosynthesis of cutin, suberin, wax, unsaturated fatty acids, and betalain (Supplementary Table S5).

### 2.5 Combined Metabolome and Transcriptome Analysis

**FIGURE 7:**
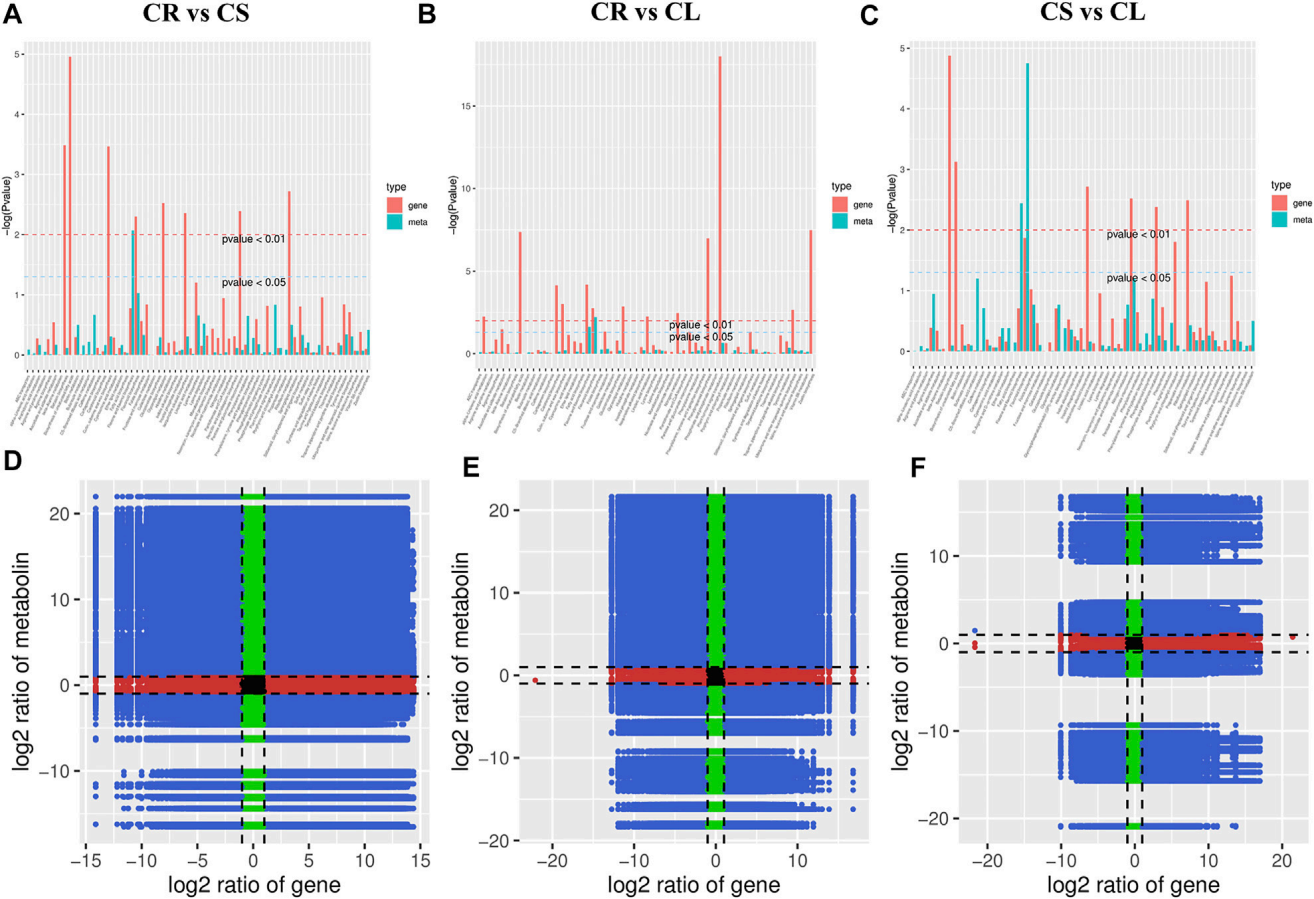
Combined analyses of differentially expressed genes and accumulated metabolites. **(A–C)** KEGG enrichment *p*-value histograms and **(D–F)** Pearson’s correlation coefficient of genes and metabolites represented as nine-quadrant flat diagrams. Black dots = unchanged genes/metabolites, green dots = DAMs with unchanged genes, red dots = DEGs with unchanged metabolites, blue dots = DEGs and DAMs. The Pearson’s correlation coefficient is > 0.8 throughout the nine-quadrant graphs.

To further understand the link between the DAMs and DEGs, we performed a co-joint analysis on both omics data. The combined KEGG pathway enrichment analysis showed that the DEGs and DAMs were enriched in multiple pathways. In particular, we noted that the DEGs and DAMs were enriched in the flavone and flavanol biosynthesis pathways (*p*-value < 0.05 and <0.01). Further, other pathways related to flavonoid biosynthesis were also enriched, further confirming the individual metabolome and transcriptome KEGG pathway enrichment results for all three comparisons (Figures 7A–C). Log2 conversion data for both metabolites and genes having PCC > 0.8 were selected and nine-quadrant diagrams were generated for each comparison (Figures 7D–F). The DEGs and DAMs in the 3^rd^ and 7^th^ quadrants for each comparison indicate that the differential expression pattern of the genes and differential accumulation of metabolites are consistent. This indicates that the changes in metabolite accumulation may be positively regulated by the genes in these quadrants. Since there are tens of pathways to which DEGs and DAMs were enriched, for brevity we have presented the results related to the flavonoid biosynthesis pathway as follows. We chose the flavonoid biosynthesis pathway due to its significant enrichment in all the tissue comparisons. Another reason for choosing this pathway for elaboration was the higher accumulation of flavonoids in leaves and stems as compared to tubers.

We found that ten DEGs (F3H, CYP73A, CCOAOMT, CHS, CHI, FLS, CYP75B1, CYP98A (C3′H), HCT, and DFR) were either positively or negatively correlated with 24 DAMs in the three comparisons (Figure 7; Supplementary Table S6). This observation indicates that the expression of multiple transcripts (or the same) of the same gene affects the accumulation of different metabolites. For example, CYP73A showed a positive correlation with 16 metabolites that were enriched in the flavonoid biosynthesis pathway. This means that the higher expression of CYP73A can lead to a higher accumulation of the positively correlated DAMs. PCC between DAMs and DEGs enriched in the flavonoid biosynthesis pathway ranging from −1 to 1 (Supplementary Table S6).

## 3 Discussion

### 3.1 *C. convolvulacea* Tubers, Stems, and Leaves Offer a Range of Compounds for Medicinal Use

Limited metabolome research on the *Codonopsis* species offers unique challenges as well as opportunities for researchers to characterize the useful compounds. Although Tibetan herbal medicine and Chinese traditional medicine have reported on the use of *C. convolvulacea* in different treatments (Guang-Xuan et al., 2001; Tang et al., 2017), it is not known which specific metabolites are present in different plant parts. Our metabolome profiling of the tuber, stem, and leaves presents interesting results that will lead toward specialized usage of these plant organs in traditional medicine. In particular, our results that the leaves and stems are a rich source of flavonoids as compared to roots are interesting because previously the above-ground parts of some *Codonopsis* species have been considered as waste. These results are consistent with the earlier report on the metabolome profile of leaves and stems of *C. pilosula* (Zeng et al., 2021). Furthermore, the usefulness of flavonoids in curing a range of diseases (Yao et al., 2004) due to their bioactivity (Robak and Gryglewski, 1996) indicates that *C. convolvulacea* plants as a whole and their parts are of interest to both the traditional and modern medicine industry. In addition to flavonoids, the stems and leaves were also rich in L-tryptophan (or its derivative compounds, e.g., tryptamine), indole, anthranilic acid, and L-phenylalanine. L-Tryptophan is an essential amino acid and is used to treat insomnia, depression, anxiety, premenstrual dysphoric disorder, and teeth grinding and is used to improve athletic performance (Friedman, 2018). This means that either the leaves and stems as a whole or their extracts can be valuable plant parts and should not be discarded. The tubers of *C. convolvulacea,* as found in our results, were rich in amino acids and derivatives, free fatty acids, organic acids, phenolic acids, saccharides, alcohols, and vitamins. This is consistent with the earlier reports on *C. lanceolata* and *C. peninsula* (Du et al., 2018; Zheng et al., 2018). The compositional analyses of the roots/tubers of these two species have also shown the higher accumulation (or the presence) of amino acids, vitamins, phenolic acids, saccharides, and alcohols. The accumulation of these compounds in excess in *C. convolvulacea* tubers indicates their potential for extraction and medicinal uses. This is probably the reason for the higher use of tubers instead of stems and leaves of *C. convolvulacea* in traditional Tibetan medicine and the northwestern United States to treat the Kashin–Beck disease (Malaisse and Mathieu, 2008). Overall, these datasets not only are useful to the public and researchers but also add resources for the policymakers to consider these traditional sources from a newer perspective within the framework of the “Healthy China 2030” (http://en.nhc.gov.cn/HealthyChinaActionPlan.html) plan of the Chinese government.

### 3.2 Flavonoid Biosynthesis and Accumulation in *C. convolvulacea*

**FIGURE 8:**
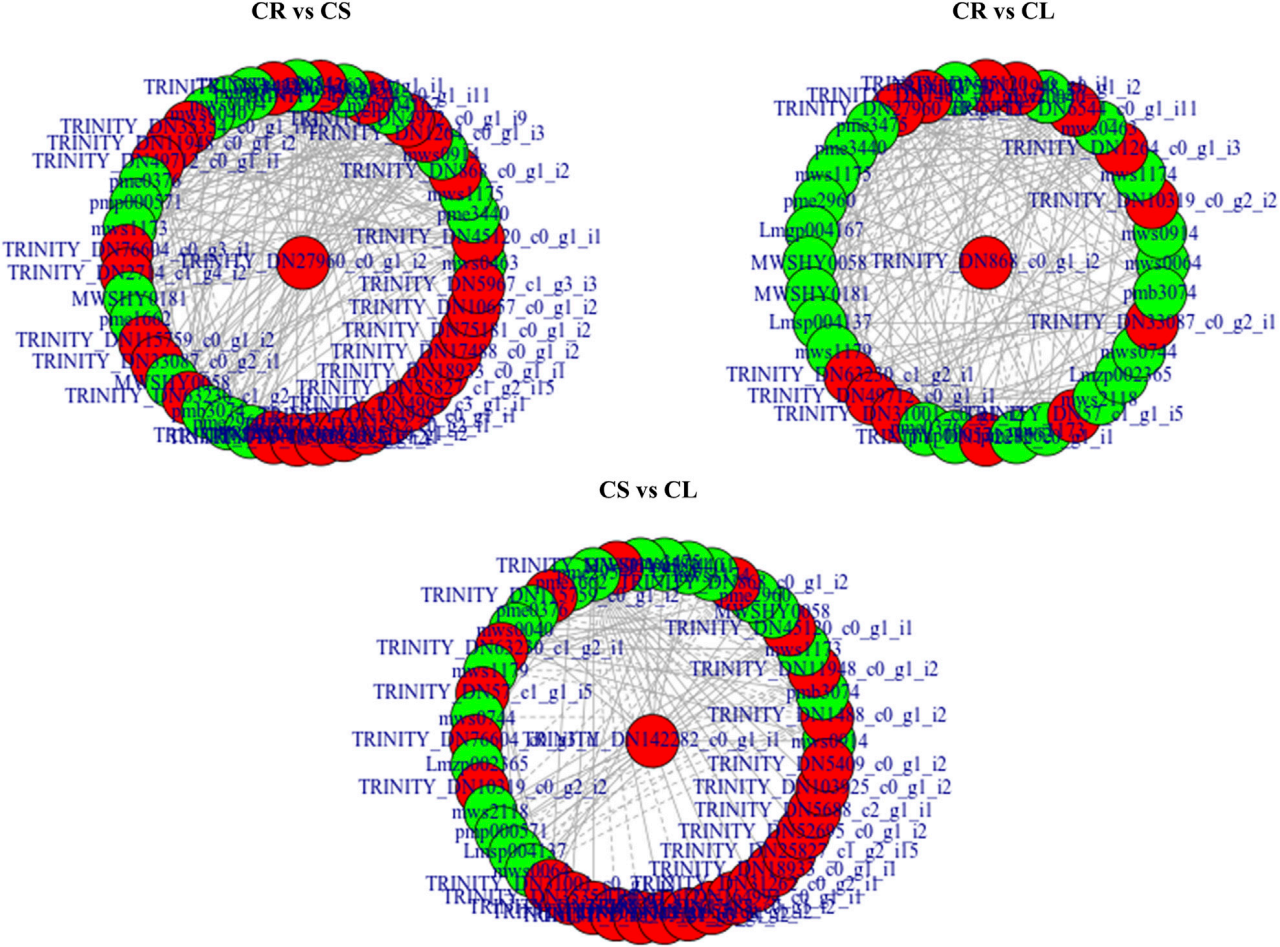
Network showing correlation (>0.8) between the DEGs and DAMs enriched in flavonoid biosynthesis pathway. Metabolites are marked in green, genes are marked in red, solid lines represent positive correlation, and dotted lines represent the negative correlation.

**FIGURE 9:**
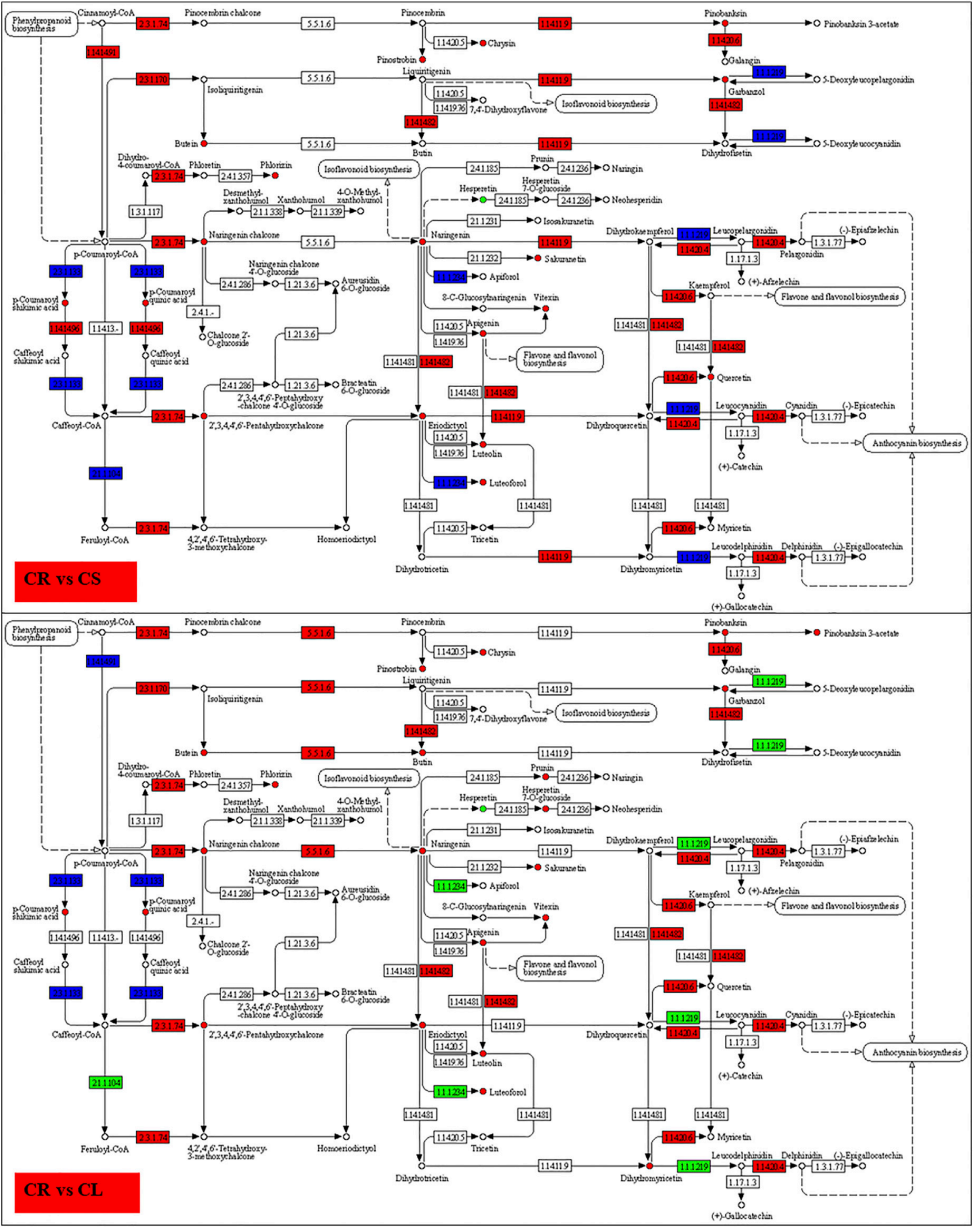
Enrichment of DEGs and DAMs on flavonoid biosynthesis pathway in CR vs. CS and CR vs. CL. The DAMs highlighted in red and green were accumulated in higher and lower quantities in CS and CL as compared to CR, respectively. The DEGs in red, green, and blue represent the genes that showed higher, lower, and mixed expression, respectively, in CS and CL as compared to CR.

Determination of the flavonoid content and the mechanisms governing their biosynthesis in dietary sources is an active topic for the last few decades. This is due to their health benefits that have been revealed through epidemiological studies (Yao et al., 2004). Their health beneficiary effects and human dietary intake as antioxidants stress the need to find novel flavonoid sources and evaluate them. Our results that the leaves, stems, and tubers have flavonoids are important from the applied and basic research perspective. Transcriptome sequencing is an effective tool to possibly understand the differential accumulation of flavonoids and identify novel candidate genes for gene manipulation. The higher accumulation of chrysin, galangin, pinobanksin, betein, phlorizin, p-coumaroyl quinic acid, luteolin, luteoforol, quercetin, vitexin, naringenin chalcone, naringenin, and sakuranetin in CL and CS as compared to CR (Supplementary Table S5) can be linked to the higher expressions of the respective genes (Figures 8 and 9). In particular, we can relate the observed changes in the isoflavone content to the expressions of CHI, CYP73A, CYP98A (C3′H), F3H, CYP75B1, ANS, and FLS. Moreover, the higher isoflavone content in CL can be related to the higher expression of CHS since it converts p-coumaroyl-CoA to isoliquiritigenin (Feinbaum and Ausubel, 1988), which is subsequently converted to different isoflavones (Du et al., 2010). In a similar way, its expression also correlates with the accumulation of naringenin chalcone and naringenin (Yahyaa et al., 2017). The higher contents of quercetin in CS and CL as compared to CR are due to the increased expressions of the ANS, FLS, CYP75B1, and the upstream genes mentioned above (Figure 9). Quercetin is synthesized from kaemferol or dihydroquercetin by the action of FLS or ANS, respectively. Functional characterization of CYP75B1 in *Camellia sinensis* has shown its role in flavonoid biosynthesis (Wang et al., 2014). On the other hand, the characterization of FLS in *Citrus unshiu* revealed its role in the conversion of dihydrokaempferol to kaempferol and dihydroquercetin to quercetin (Wellmann et al., 2002). Based on these results, we can propose that the expression of flavonoid biosynthesis-related genes, i.e., CHI, CHS, CYP73A, CYP98A, C3′H, F3H, CYP75B1, ANS, and FLS is important and these genes should be characterized under different growing conditions and at different plant stages to determine the most suitable harvesting time to achieve higher flavonoid production.

### 3.3 Tryptophan Biosynthesis in *C. convolvulacea*

Tryptophan, being an essential plant-derived amino acid, has significant value in human nutrition. Tryptophan and the bioactive metabolites produced from it have the potential to contribute to a broad range of health conditions, e.g., autism, cardiovascular problems, depression, multiple sclerosis, kidney diseases, and cognitive function (Friedman, 2018). Apart from these functions, tryptophan also shows antimicrobial, antidiabetic, and anti-inflammatory functions (Nayak and Buttar, 2015). Thus, finding novel plant sources for tryptophan is an ongoing task. Our findings that CL had higher indole accumulation followed by CS and CR are interesting and provide information on the use of specific tissues. It is a product of the action of tryptophanase (DDC) on L-tryptophan (Snell, 1975) and this could be due to the upregulation of one DDC (*TRINITY_DN5862_c0_g1_i10*) in leaves as compared to CS. Tryptophan is also converted into tryptamine by the action of DDCs. The highest accumulation of tryptamine in CS can be linked to the sole expression of five TDC/DDCs (aromatic-L-amino-acid/L-tryptophan decarboxylase; *TRINITY_DN50728_c0_g1_i2, TRINITY_DN68814_c0_g2_i1, TRINITY_DN68814_c0_g1_i1, TRINITY_DN178280_c0_g1_i1*, and *TRINITY_DN79687_c0_g2_i2*). This is consistent with the long-known role of TDC/DDC when expressed in tobacco (Songstad et al., 1990). Not only these but also the genes present in the upstream pathways, i.e., photosynthesis and phenylpropanoid biosynthesis pathways, are also a possible possibility to reason for the higher accumulation of most of the metabolites enriched in the tryptophan metabolism pathway. Research on the exogenous application of tryptophan (and other amino acids) improves photosynthetic assimilation (Khan et al., 2019). However, understandably, higher photosynthesis would result in higher biosynthesis of tryptophan or other related metabolites but specific research on *C. convolvulacea* would reveal further details. The genes enriched in photosynthesis and phenylpropanoid biosynthesis pathways are ideal candidates for higher tryptophan accumulation. Furthermore, it would be possible to avoid/reduce tryptophan conversion to indole and tryptamine by downregulating DDCs and TDC/DDCs in leaves and stems.

### 3.4 Alkaloid Biosynthesis in *C. convolvulacea*

To develop a highly diversified medicine portfolio for human and animal use, plant-based natural products offer a useful resource (Ganesan, 2008). In this regard, alkaloids play a dual role, i.e., *in-planta* they protect plants from invading pathogens as well as regulate growth, whereas they offer a range of health benefits (Kurek, 2019). Our findings that both the *C. convolvulacea* leaves and stems had a higher accumulation of alkaloids are interesting from the usage point of view. Besides, these two plant parts appear a viable raw material for the extraction of alkaloids and other metabolites accumulated in them for the commercial medicine industry. Stem-specific expression of a large number of DDCs indicates that L-tyrosine, in the stem, is being converted to tyramine and dopamine. It is known that DDCs are also responsible for these conversions apart from their role in tryptamine biosynthesis as reported in the above section (Facchini et al., 2000). The expression of DDCs and higher concentrations of tyramine in CS are consistent (Table 1; Supplementary Table S5). The higher expressions of ASP5 (De la Torre et al., 2014), ARO8 (Hirata et al., 2012), GOT, (Murooka et al., 2002), and AOC3 further indicate the conversion of L-tryptophan and L-tyrosine to downstream metabolites (Tipping and Mcpherson, 1995). Relative to lower expressions of these genes in CL are consistent with the respective metabolite accumulation as compared to CS. From these observations, we can understand that leaves could be a usable source for alkaloids in addition to the compounds discussed in the above sections, i.e., flavonoids. The stem also may act as an additional source of alkaloids and flavonoids. But it is also important to keep in mind that in the stem, these compounds might have been detected as they were being transported from tuber to leaves or vice versa. In contrast, CS specific expression of a large number of DEGs, e.g., HPD, 4CL, wrbA, HPDs, CYP73A, and ARO8 (Supplementary Table S5), excludes this assumption and suggests biosynthesis or interconversion of these compounds in the stem. However, these assumptions need further research.

### 3.5 Future Research on *C. convolvulacea*

*C. convolvulacea* and other species in this genus have long been used in traditional Chinese and Tibetan medicine (Hong, 2015). Our metabolome results highlight the significance of *C. convolvulacea* from traditional medicine’s point of view as well as from its utility as a raw material in the plant-based commercial production of flavonoids, tryptophan, amino acids, and alkaloids. However, since this research is a pioneering attempt in this species, many questions should be answered for its utility. In particular, future studies should focus on the age-specific detection of metabolites in different plant parts and how these metabolites accumulate with the aging of the plants. Furthermore, differential accumulation of these metabolites would need studies focusing on gene-specific characterization experiments to elucidate the roles of individual genes. Since commercial use of this species as raw material would need its cultivation on farmland, it would be interesting to know how the accumulation of different compounds will be affected under different growing conditions and biotic and abiotic stresses. An interesting research avenue could be finding ways to enhance the biosynthesis of compounds of interest. One strategy could be the application of abiotic stress at specific plant growth stages. One approach, which has been investigated in different plant species, could be the increasing endogenous plant hormone signaling and/or exogenous application of specific hormones (Ji et al., 2019). Likewise, it is known that abiotic stresses result in the increased accumulation of alkaloids and other metabolites (Kumar and Sharma, 2018; Jan et al., 2021). Can we use a similar strategy in *C. convolvulacea*? From these perspectives, our combined metabolome and transcriptome study provides an initial but detailed understanding of the pathways related to alkaloids, flavonoids, tryptophan, and other metabolites.

## 4 Conclusion

This is the first detailed combined transcriptome and metabolome study on the three *C. convolvulacea* tissues, i.e., tuber, stem, and leaf. We conclude that the three tissues contain a useful variation of a range of compounds classified as flavonoids, alkaloids, phenolic acids, and amino acids and derivatives. We found that the stem and leaf are rich in flavonoids and alkaloids, whereas the tuber is a rich source of amino acids and derivatives. The KEGG pathway enrichment revealed the role of DEGs enriched in flavonoid biosynthesis, phenylpropanoid biosynthesis, tryptophan metabolism, indole alkaloid biosynthesis, and cutin, subarin, and wax biosynthesis pathways. We conclude that this study provides useful knowledge on the utility of the tuber, stem, and leaf in traditional as well as modern medicine.

## 5 Materials and Methods

We selected natural plants of *C. convolvulacea* Kurz var. vinciflora (Kom.) L.T. Shen that showed consistent growth potential and lacked any pathogen or insect attack, as the study material. The selected plants were harvested in Nyingchi (N: 29°40′23.40″, E: 94°20′28.28″), Tibet Autonomous Region, China in September 2021 (Zichao, 2007). Plants showing similar growth stages were chosen for metabolome and transcriptome analysis. Three *C. convolvulacea* tissues, i.e., tubers (CR), leaves (CL), and stems (CS), were separated from the selected triplicate plants (Supplementary Figure S4). The harvested tissues were washed thoroughly under tap water followed by washing thrice with double-distilled water. The excess water was removed by drying on a paper towel followed by freezing in liquid nitrogen and storing at −80°C until being processed further for analyses.

### 5.1 Metabolome Analysis

#### 5.1.1 Sample Preparation and Extraction

Triplicate samples of tubers, shoots, and leaves were freeze-dried using a vacuum freeze-dryer and crushed in a mixer mill (MM 400, Retsch) using zirconia beads for 1.5 min at 30 Hz. The CR, CS, and CL samples were processed separately. Once the tissues were crushed to powder, a 100 mg sample was dissolved in 70% methanol (1.2 ml) by vortexing for 30 s after every half an hour. This process was repeated six times followed by placing the extract in a refrigerator at 4°C overnight. The extracts were then centrifuged at 12,000 rpm for 10 min, filtered through a 0.22 μm pore size filter (ANPEL, Shanghai, China), and then processed for UPLC-MS/MS analysis.

#### 5.1.2 UPLC Conditions and ESI-Q TRAP-MS/MS

The sample extracts were analyzed using a UPLC-ESI-MS/MS system (UPLC, SHIMADZU Nexera X2, https://www.shimadzu.com.cn/; MS, Applied Biosystems 4500 Q TRAP, https://www.thermofisher.cn/cn/zh/home/brands/applied-biosystems.html). The UPLC analysis conditions were as reported earlier (Zhou et al., 2020). The linear ion trap (LIT) and QQQ (triple quadrupole) were obtained by using a QQQ-LIT mass spectrometer, AB4500 Q TRAP UPLC/MS/MS System. The system was equipped with an ESI Turbo Ion-Spray interface, which operated in both + and −ion modes. This operation was controlled by Analyst 1.6.3 software (AB Sciex). The source operation parameters for ESI, UPLC/MS/MS measurements and data collections/conversions were done as reported earlier (Zhou et al., 2020).

#### 5.1.3 Metabolome Data Analysis

First, we unit variance-scaled the data and then computed an unsupervised PCA using prcomp within R (www.r-project.org). PCC between the samples were computed using the cor function in R. Furthermore, we determined the significantly regulated metabolites by computing VIP ≥ 1 and absolute log2 FC (fold change) ≥ 1. The VIP values ​​were extracted from the OPLS-DA result that was generated using the R package MetaboAnalystR. The data were log transformed (log2) and the mean centered before OPLS-DA. To avoid overfitting, a permutation test (200 permutations) was performed. The detected metabolites were annotated using the KEGG Compound database (http://www.kegg.jp/kegg/compound/) followed by mapping of the DAMs on KEGG pathways in the KEGG pathway database (http://www.kegg.jp/kegg/pathway.html). Pathways with significantly regulated metabolites mapped to were then fed into metabolite sets enrichment analysis, and their significance was determined by the hypergeometric test’s *p*-values.

### 5.2 Transcriptome Analysis

#### 5.2.1 RNA Extraction, Library Preparation, and Machine Sequencing

Total RNAs were extracted from the triplicate samples of CS, CL, and CR separately and magnetic beads with Oligo (dT) were then used to enrich the mRNAs. The mRNAs were broken into short fragments using fragmentation buffer and were then used as a template to synthesize the first-strand cDNA with random hexamers. The second strand was then synthesized by adding buffer, dNTPs, RNase H, and DNA polymerase I, followed by purification of cDNA by the QiaQuick PCR kit and elution with EB buffer. The ends are repaired, poly(A) tailed, and the sequencing adapter was connected. We then performed fragment size selection by agarose gel electrophoresis, and finally, PCR amplification was performed. The separate libraries were then (pair-end) sequenced on Illumina HiSeq™.

#### 5.2.2 Computational Analysis of the Transcriptome Sequencing Data

First of all, we processed the raw data to obtain clean data. For this, we removed the reads having adaptors, or if the paired reads had N content >10% or if the low-quality basis (Q ≤ 20) in sequencing reads exceeded 50%. Furthermore, we determined the error distribution as well as the GC content of the raw reads. For quality control analyses, FastQC was used (Andrews, 2017).

Once we obtained the clean reads, transcripts were assembled using Trinity (2.6.6, (Grabherr et al., 2011)) and unigenes were identified. The unigenes were then used for BLAST (Johnson et al., 2008) to compare the unigene sequences with different databases including KEGG (Kanehisa and Goto, 2000), NR (Deng et al., 2006), Swiss-Prot (Apweiler, 2001), GO (Ashburner et al., 2000), COG/KOG (Koonin et al., 2004), and Trembl databases (Apweiler et al., 2004). Apart from these databases, we also compared the sequences with Pfam after predicting unigene amino acid sequences (Bateman et al., 2002). After BLAST, the expression of the genes was quantified. For this, first, the spliced transcripts by Trinity were used as a reference sequence and the clean reads of each sample were mapped to it by using bowtie2 (Langmead and Salzberg, 2012) in RSEM (Li and Dewey, 2011). This was followed by the calculation of FPKM. For visualization of the FPKM values, we used R to prepare box plots. PCA and PCC were then computed using the expression data for each tissue type. The DEGs between the three types of tissues were found using DESeq2 (Varet et al., 2016). We then used the Benjamini–Hochberg method (Benjamini and Hochberg, 1995) to perform hypothesis test correction on *p*-value and obtained a false discovery rate (FDR) followed by the screening of the DEGs based on FDR (<0.05) as well as |log2 foldchange| ≥1. Venn diagrams were prepared in InteractiVenn (Heberle et al., 2015).

The DEGs were then mapped onto KEGG pathways in KOBAS2.0 (Xie C et al., 2011) and the FDR (<0.05) was used to reduce the false-positive prediction of enriched KEGG pathways. Three parameters, i.e., Rich factor, Q-value, and the number of DEGs on each pathway were considered for the degree of KEGG enrichment. The transcription factor prediction was done in iTAK software (Zheng et al., 2016).

### 5.3 Co-Joint Analyses

To determine the links between transcriptome and metabolome datasets, we also analyzed both results jointly. First, we performed a combined KEGG pathway enrichment analysis to determine the degree of enrichment of KEGG pathways. We then used the Corson program in R to calculate PCC between DEGs and DAMs. The PCC was then used to construct the transcript–metabolite network.

## Data Availability

The datasets presented in this study can be found in online repositories. The names of the repository/repositories and accession number(s) can be found in the article/Supplementary Material.
