# Supplementary material for: Transcriptome and Metabolome Analyses of Codonopsis convolvulacea Kurz Tuber, Stem, and Leaf Reveal the Presence of Important Metabolites and Key Pathways Controlling Their Biosynthesis
Source: Front Genet. 2022 Jul 25;13:884224. doi: 10.3389/fgene.2022.884224 (PMC9359469; doi:10.3389/fgene.2022.884224)
Supplement: Supplementary file 1 [file Presentation1.PPTX]

## Slide 1
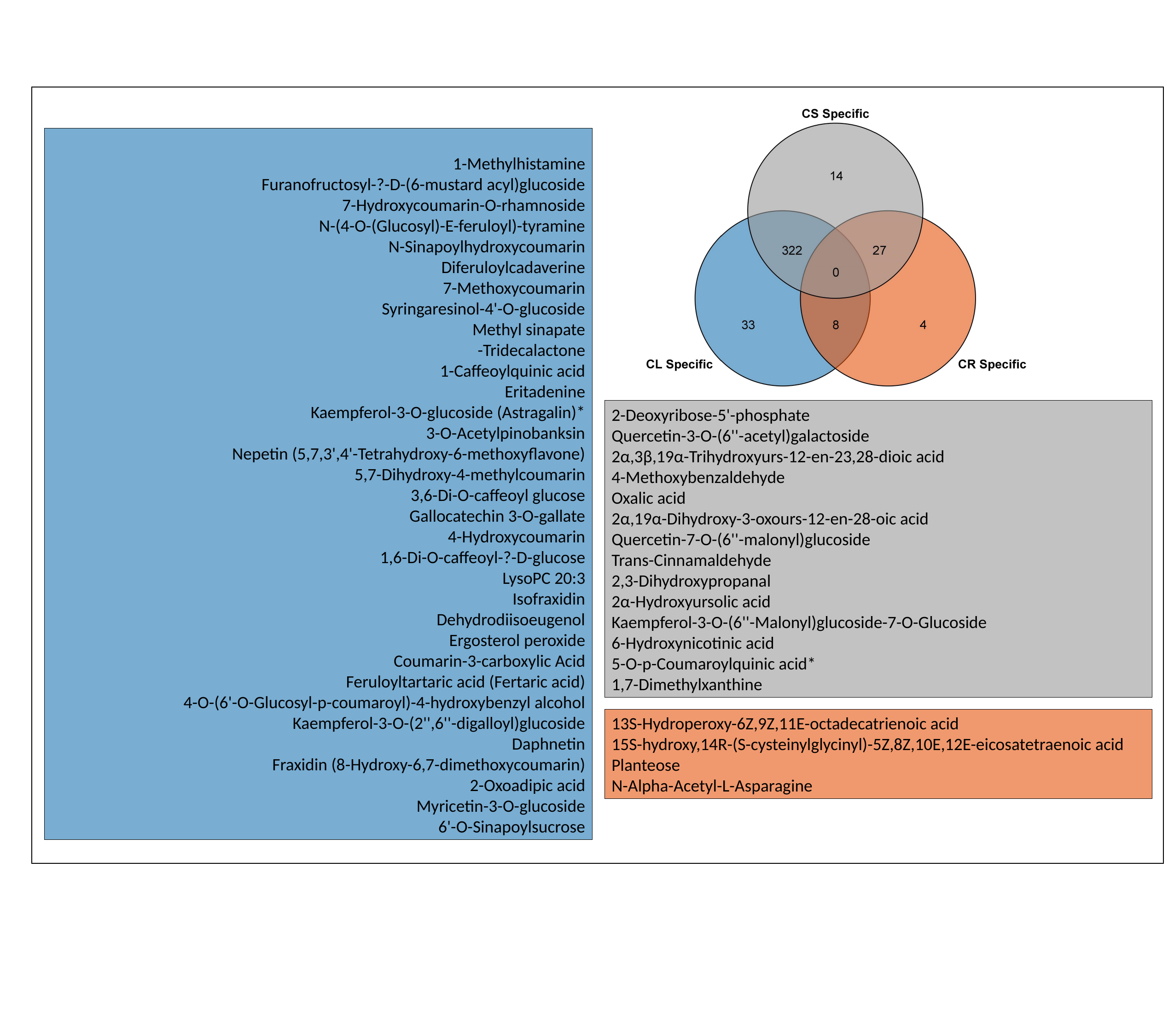

1-Methylhistamine
Furanofructosyl-?-D-(6-mustard acyl)glucoside
7-Hydroxycoumarin-O-rhamnoside
N-(4-O-(Glucosyl)-E-feruloyl)-tyramine
N-Sinapoylhydroxycoumarin
Diferuloylcadaverine
7-Methoxycoumarin
Syringaresinol-4'-O-glucoside
Methyl sinapate
-Tridecalactone
1-Caffeoylquinic acid
Eritadenine
Kaempferol-3-O-glucoside (Astragalin)*
3-O-Acetylpinobanksin
Nepetin (5,7,3',4'-Tetrahydroxy-6-methoxyflavone)
5,7-Dihydroxy-4-methylcoumarin
3,6-Di-O-caffeoyl glucose
Gallocatechin 3-O-gallate
4-Hydroxycoumarin
1,6-Di-O-caffeoyl-?-D-glucose
LysoPC 20:3
Isofraxidin
Dehydrodiisoeugenol
Ergosterol peroxide
Coumarin-3-carboxylic Acid
Feruloyltartaric acid (Fertaric acid)
4-O-(6'-O-Glucosyl-p-coumaroyl)-4-hydroxybenzyl alcohol
Kaempferol-3-O-(2'',6''-digalloyl)glucoside
Daphnetin
Fraxidin (8-Hydroxy-6,7-dimethoxycoumarin)
2-Oxoadipic acid
Myricetin-3-O-glucoside
6'-O-Sinapoylsucrose
2-Deoxyribose-5'-phosphate
Quercetin-3-O-(6''-acetyl)galactoside
2α,3β,19α-Trihydroxyurs-12-en-23,28-dioic acid
4-Methoxybenzaldehyde
Oxalic acid
2α,19α-Dihydroxy-3-oxours-12-en-28-oic acid
Quercetin-7-O-(6''-malonyl)glucoside
Trans-Cinnamaldehyde
2,3-Dihydroxypropanal
2α-Hydroxyursolic acid
Kaempferol-3-O-(6''-Malonyl)glucoside-7-O-Glucoside
6-Hydroxynicotinic acid
5-O-p-Coumaroylquinic acid*
1,7-Dimethylxanthine
13S-Hydroperoxy-6Z,9Z,11E-octadecatrienoic acid
15S-hydroxy,14R-(S-cysteinylglycinyl)-5Z,8Z,10E,12E-eicosatetraenoic acid
Planteose
N-Alpha-Acetyl-L-Asparagine
