# Supplementary figures and images for: Transcriptome and Metabolome Analyses of Codonopsis convolvulacea Kurz Tuber, Stem, and Leaf Reveal the Presence of Important Metabolites and Key Pathways Controlling Their Biosynthesis
Source: Front Genet. 2022 Jul 25;13:884224. doi: 10.3389/fgene.2022.884224 (PMC9359469; doi:10.3389/fgene.2022.884224)

## Slide 1
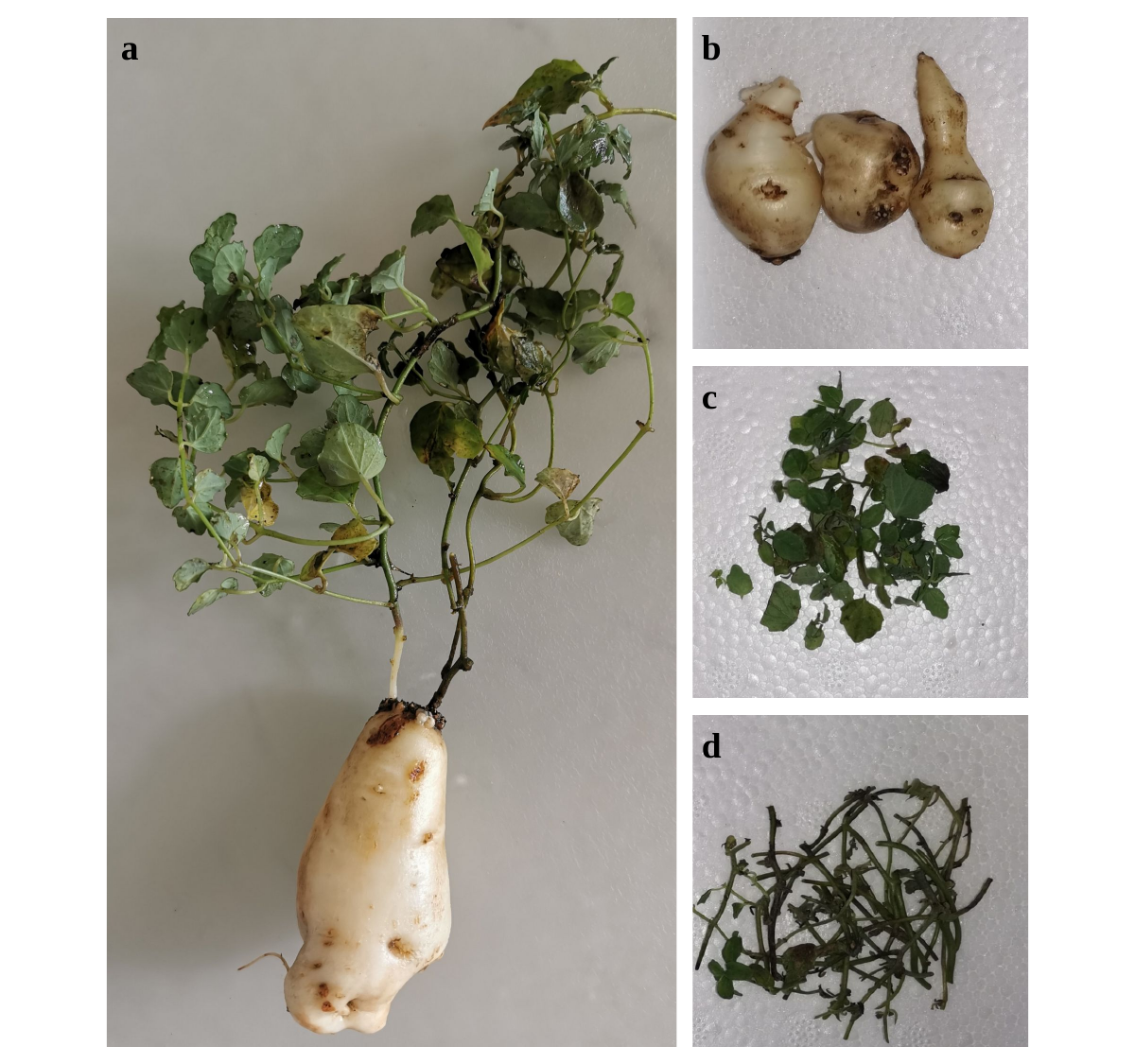

a
b
c
d

Supplement: Supplementary file 2 [file Presentation4.PPTX]

## Slide 1
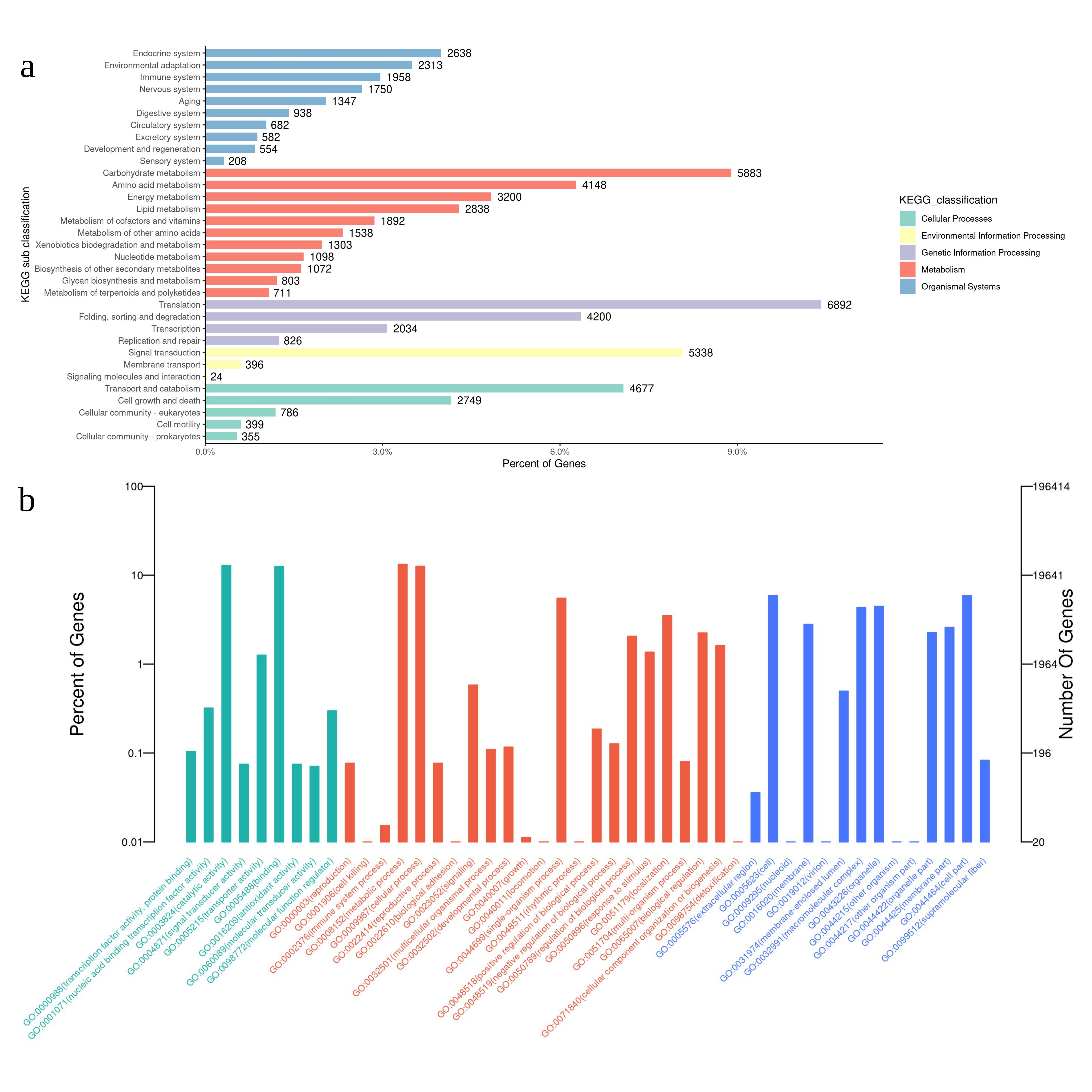

a
b

Supplement: Supplementary file 3 [file Presentation3.PPTX]

## Slide 1
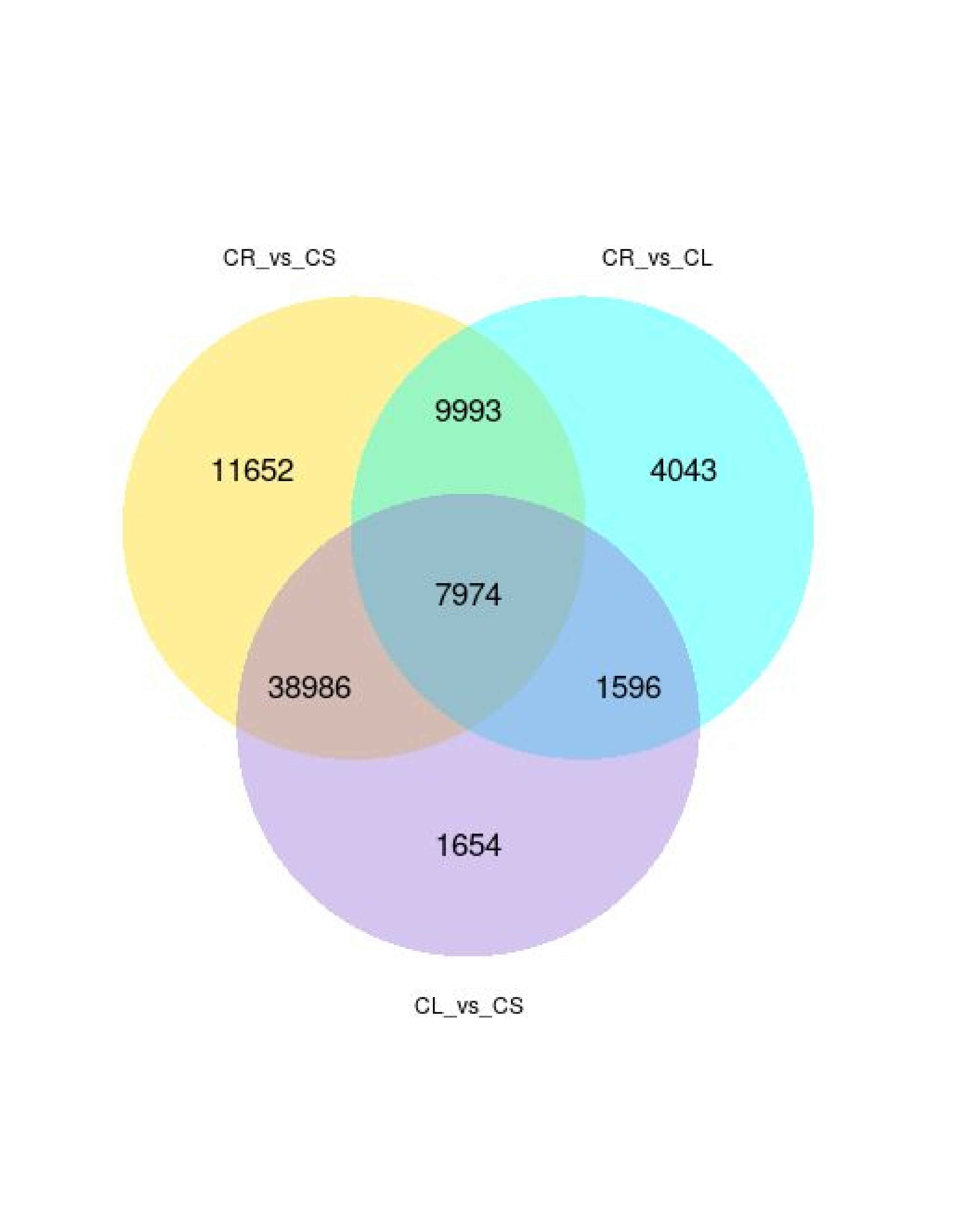

Supplement: Supplementary file 4 [file Presentation2.PPTX]
